# 

**Published:** 1889-04-06

**Authors:** 


PRICE ONE PENNY.
No. 132, Vol. VI.—April 6th, 1889.
Registered at the General Post Office as a Newspaper.]

Per Annum, 6s. 6d., post free.

Monthly Parts, 6d.; post free, 7½d.

Ditto per Annum, 7s. 6d., post free.

